# Treating nightmares in posttraumatic stress disorder with dronabinol: study protocol of a multicenter randomized controlled study (THC PTSD-trial)

**DOI:** 10.1186/s12888-023-04818-5

**Published:** 2023-05-05

**Authors:** Stefan Roepke, Nikola Schoofs, Kathlen Priebe, Felix Wülfing, Christian Schmahl, Robert Röhle, Jenny Zähringer, Tobias Lotter, Christian Otte, Stefanie Koglin

**Affiliations:** 1 Department of Psychiatry and Neurosciences, Charité - Universitätsmedizin Berlin, Campus Benjamin Franklin, Hindenburgdamm 30, 12203 Berlin, Germany; 2 Oberberg Fachkliniken for Psychiatry, Psychosomatics and Psychotherapy, Berlin and Brandenburg, Germany; 3 Department of Psychiatry and Neuroscience, Charité Universitätsmedizin Berlin, Charité Campus Mitte, Berlin, Germany; 4 Department of Psychosomatic Medicine and Psychotherapy, Central Institute of Mental Health, Medical Faculty Mannheim/Heidelberg University, Mannheim, Germany; 5 Charité – Universitätsmedizin Berlin, Institute of Biometry and Clinical Epidemiology, Berlin, Germany; 6 Department of Psychosomatic Medicine and Psychotherapy, University Medical Centre Hamburg-Eppendorf, Hamburg, Germany

**Keywords:** Clinical trial protocol, Randomized clinical trial, Dronabinol, BX-1, Posttraumatic stress disorder, Nightmares

## Abstract

**Background:**

Distressing nightmares are a core symptom of posttraumatic stress disorder (PTSD) and contribute to psychiatric comorbidity, impaired physical health and decreased social functioning. No specific pharmacological treatment for PTSD-related nightmares is yet approved. Preliminary clinical data indicate that cannabinoid agonists can improve nightmares and overall PTSD symptoms in patients with PTSD. The primary objective of the study is to examine the efficacy of oral dronabinol (BX-1) versus placebo in reducing nightmares in patients with PTSD. The secondary objectives of the study are to examine the efficacy of oral BX-1 in reducing other PTSD symptoms.

**Methods:**

The study is designed as a multi-centric, double-blind, randomized (1:1), placebo-controlled, parallel group interventional trial. Eligible patients will be randomized to BX-1 or placebo, receiving a once-daily oral dose before bedtime for 10 weeks. Primary efficacy endpoint is the Clinician-Administered PTSD Scale (CAPS-IV) B2 score for the last week, measuring frequency and intensity of nightmares. Secondary efficacy endpoints are other disorder-specific symptoms in patients with PTSD. Further, tolerability and safety of dronabinol will be assessed.

**Discussion:**

This randomized controlled trial will provide evidence whether treating patients with PTSD and nightmares with dronabinol is safe and efficacious.

**Trial registration:**

NCT04448808, EudraCT 2019–002211-25.

**Supplementary Information:**

The online version contains supplementary material available at 10.1186/s12888-023-04818-5.

## Background and rational

Posttraumatic stress disorder (PTSD) is associated with serious disability, medical illness, and a severely impaired quality of life [14, 20]. The WHO has reported an overall disability-adjusted life year (DALY) rate per 100,000 inhabitants of Germany of 54 for PTSD; 80 in women, 28 in men [22]. PTSD places a high socioeconomic burden on patients with marked reductions in household income [19]. More than 50% of patients with PTSD suffer from comorbid mood, anxiety, and substance-use disorders [16]. Various medical comorbidities are very common in PTSD patients with cardiovascular diseases among the most prevalent [15]. Nightmares also contribute to alcohol and substance abuse, suicidal ideation, and even completed suicide [17]. Further, distress related to insomnia is one of the most common residual symptoms after specific psychotherapy for PTSD [13]. Only selective serotonin reuptake inhibitors (SSRIs; in Germany and FDA: paroxetine and sertraline) are currently approved as pharmacological treatment for PTSD with low response and remission rates [10] and there is no evidence that SSRIs have an effect on nightmares in PTSD [7].

Accumulating clinical data indicate that cannabinoid agonists (e.g., dronabinol) can improve nightmares and overall PTSD symptoms in patients with PTSD [23]. First, many patients with PTSD cite motives of self-medication for continued use of cannabis due to its ability to promote relaxation and sleep, and reduce anxiety symptoms and hyperarousal [2, 5, 6]. Second, an uncontrolled open-label study of nabilone (0.5-6 mg/day) at bedtime reduced nightmares in patients with PTSD, 34 out of 47 patients exhibiting either total cessation or significant reduction in nightmares [11]. Third, a retrospective chart review showed that nabilone (mean dose 4 mg/day) treatment was associated with a significant improvement in sleep and a reduction in nightmares (severity and frequency) as well as a general reduction in PTSD symptom severity [8]. Fourth, an open label, uncontrolled pilot study examined the impact of adding 5 mg of Δ(9)-THC twice a day as add-on treatment onto existing medications in patients with PTSD and found that THC consumption specifically improved sleep quality, reduced nightmares, and reduced symptoms of hyperarousal [18]. Fifths, a small, randomized, double-blind, placebo controlled crossover study assessed nabilone (3 mg daily) and found significant reduction in severity and frequency of nightmares, and increased general well-being [12]. After approval of the first version of this study protocol (version 1.1), results of a randomized trial on smoked cannabis preparations versus placebo on symptoms in patients with PTSD was published [4]. Results revealed no significant difference in change in PTSD symptom severity between the active cannabis concentrations and placebo. However, the small sample size (*N* = 20 per group) and short observation period (3 weeks) restrict the interpretation of the findings. Nevertheless, based on these findings, the sponsor of the current study decided to implement an initially unplanned interim analysis (see statistical analysis plan for interim analysis in the supplements, description in the manuscript and in protocol version 2.0).

### Objectives

The primary objective of the study is to examine the efficacy of oral BX-1 over placebo in reducing nightmares in patients with PTSD. The secondary objectives of the study are to examine the efficacy of oral BX-1 in reducing other PTSD-specific symptoms.

### Trial design

The THC-PTSD-Study is designed as a multi-centric, double-blind, randomized (1:1), placebo-controlled, parallel group interventional exploratory phase II trial.

## Methods

The protocol follows the Standard Protocol Items: Recommendations for Interventional Trials (SPIRIT) 2013 statement [9]. Corresponding sections are outlined in the SPIRIT checklist (see Supplement).

### Study sample and setting

**Fig. 1:**
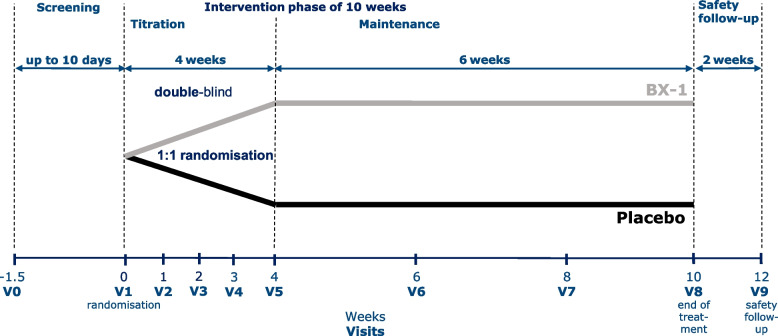
Treatment schedule

One hundred seventy-six patients with nightmares and PTSD will be enrolled in the study (but see above for interim analysis). Participants will be allocated to two treatment arms: oral dronabinol (BX-1) or placebo (see Fig. 1).

The study will be conducted at three university hospitals in Germany which provide specialized PTSD treatment services: The Department of Psychiatry and Neuroscience at Charité – Universitätsmedizin Berlin, Campus Benjamin Franklin, the Department of Psychiatry and Neuroscience, Charité – Universitätsmedizin Berlin at St. Hedwig Hospital, and the Department of Psychosomatic Medicine and Psychotherapy, Central Institute of Mental Health Mannheim.

### Eligibility criteria

Patients must provide written, informed consent before any study procedures occur (see supplements for Informed Consent Form). Patients eligible for the trial must comply with the inclusion criteria (see Table 1) at randomization. Exclusion criteria are listed in Table 1.

**Table 1:** In- and exclusion criteria of the THC-PTSD-trial

Inclusion criteria	Exclusion criteria
1. Diagnosis of posttraumatic stress disorder (PTSD) according to DSM-5 with a 20 item CAPS-5 total score ≥ 26	1. Lifetime cannabis use disorder
2. At least two nightmares a week, an intensity score ≥ 2, with a CAPS-IV B2 (frequency and intensity for the last week) score ≥ 5	2. Current substance/alcohol use disorder (≤ 3 months)
3. Men and women between 18 and 65 years of age	3. Acute suicidality
4. Written informed consent	4. Psychotic disorder
5. The patient has the capacity to give consent (He/she is able to understand the nature and anticipated effects/side effects of the proposed medical intervention)	5. Bipolar disorder
6. The patient is not breastfeeding	6. Current anorexia nervosa
7. Women of child-bearing potential must have a negative urine or serum pregnancy test	7. Current major depressive episodes and a MADRS score > 29
8. All participants must use highly effective contraception	8. Dementia
9. The patient received stable pharmacological medication for at least 4 weeks prior to study entry (any changes in medication dose or frequency of therapy must be answered with no)	9. Trauma-focused psychotherapy four weeks before the trial
	10. Initiation of sleep medication 4 weeks prior screening or initiation of alpha adrenergic agents 4 weeks prior to screening
	11. Acute or unstable medical illness
	12. Epilepsy
	13. Relevant heart diseases
	14. Known HIV- and/or active Hepatitis-B- or Hepatitis-C-infection
	15. Current or past malignant illness
	16. The patient is unwilling to consent to saving, processing and propagation of pseudonymized medical data for study reasons
	17. Patients, who may be dependent on the sponsor, the investigator or the trial sites, have to be excluded from the trial
	18. The patient is legally detained in an official institution
	19. The patient does have a known allergy or contraindication against Dronabinol
	20. The patient does have clinically significant abnormalities in 12-lead ECG
	21. The patient does have clinically significant laboratory abnormalities
	22. The patient did participate in other interventional trials during the 3 months before and at the time of this trial

*CAPS-5* Clinician-Administered PTSD Scale for DSM-5 (last month version), *CAPS-IV B2* B2 score of the Clinician-Administered PTSD Scale for DSM-IV (frequency and intensity of nightmares), *MADRS* Montgomery–Åsberg Depression Rating Scale, *HIV* Human immunodeficiency virus, *ECG* Electrocardiogram

### Interventions

Eligible patients will be randomised in equal proportions to dronabinol (BX-1) and placebo, receiving a once-daily oral dose before bedtime for 10 weeks. Of these 10-weeks, the first 4 weeks are considered the titration period, the following 6 weeks are the maintenance period with stable dose.

BX-1 is an oily solution containing 25 mg/ml dronabinol. Dronabinol ((-) trans-Δ9-Tetra-hydrocannabinol, Δ9-THC) is the major psychoactive constituent of Cannabis sativa L.. Dronabinol is a clear to amber resin. It is highly lipid-soluble, thermolabile, photolabile and susceptible to oxidation. There is no discernible difference between BX-1 and the corresponding placebo with regard to color, taste, and appearance, thus ensuring the trial`s double-blind character. BX-1 and placebo are filled in bottles. Study medication (IMP) and placebo will be provided by Bionorica SE, Neumarkt, Germany.

### Titration period

The titration period lasts 4 weeks and is performed double-blinded, neither the patient nor the investigator/sponsor knows the identity of the IMP.

All patients in the active treatment group start with 2.5 mg BX-1 and keep the dose constant for 4 days. On the following day (day 5), an increase with additional 2.5 mg BX-1 (5 mg BX-1 total dose) can be done. This increase with additional 2.5 mg BX-1 can be repeated on day 8, day 12, day 15 and day 19 (Fig. 1). However, 15 mg BX-1 as the maximum dose per day is not the treatment goal of this trial, but patients are instructed to define the optimal dose together with the study personnel. The optimal dose is reached if nightmares are eliminated completely. Patients in the placebo condition receive an equal amount of undistinguishable placebo.

### Maintenance period

The individually established daily dose at day 28, will be maintained throughout the 6 weeks double-blinded maintenance and treatment period (Fig. 1).

### End-of study and safety follow-up

End-of-study is after 10 weeks of intervention (visit 8). No wash-out phase will be performed. Two weeks after end-of-study a safety follow-up will be performed (week 12, visit 9) (Fig. 1). No primary or secondary outcome will be assessed at safety follow-up.

### Modifications

Any modification of the dose of study medication during the trial other than described for the titration and maintenance period will be considered as protocol deviation. Participants have the right to withdraw from the study at any time and for any reason without sanction, penalty, or loss of benefits to which the subject is otherwise entitled. The investigator may decide to withdraw a subject from the study at any time with a reasonable rationale.

### Adherence

Face-to-face adherence reminders will take place at the initial product-dispensing visit and each study visit thereafter. Compliance will be checked for each patient at Visits 2–8 by weighing the bottles when returned by the patient with a calibrated balance. The compliance of the patient will be calculated for each visit and for the complete trial. Patients will be defined non-compliant for the complete trial if less than 80% or more than 120% of the individually established dose (Visit 5) was taken.

### Pharmacotherapy

All psychotropic medication needs to be stable at least 4 weeks prior to the start of the study (screening) and has to remain stable until visit 8 (end of study). All other non-psychiatric medication has to be stable at least 4 weeks prior to screening and during the entire trial with some exceptions. Medications for minor transient non-psychiatric medical conditions (e.g., common cold and menstrual cramps) such as NSAIDs are allowed. All clinical exacerbation requiring prescription of other psychotropic drugs will be considered a serious adverse event (SAE).

Concomitant pharmacotherapy with Cannabis-based medicine, Cannabis herbs, sleep medication, and alpha-adrenergic agents is not permitted. A more detailed description can be seen in the supplement section.

### Psychotherapy

Non-trauma-focused psychotherapy is allowed if initiated 2 weeks prior to screening and at stable dose during the trial. Psychotherapy frequency has to be documented in the medical file and eCRF. Trauma-focused psychotherapy is not allowed up to 4 weeks prior to the trial (screening) and during the entire trial.

### Outcomes

#### Primary endpoint

The Clinician-Administered PTSD Scale (CAPS-IV) B2 score measuring frequency and intensity of nightmares for the last week, range 0–8 [3] directly after last intervention (10 weeks).

#### Secondary endpoints

1. Change from baseline of the frequency and intensity of nightmares, measured with the Clinician-Administered PTSD Scale-IV (CAPS-IV) B2 score for the last week, range 0–8, at Visit 3 – Visit 7
2. Change from baseline of the CAPS-5 total score (overall PTSD symptoms, last week) at Visit 6 and Visit 8
3. Change from baseline of the Pittsburgh Sleep Quality Index-Addendum for PTSD (PSQI-A) (PTSD related sleep symptoms) at Visit 2 – Visit 8
4. Change from baseline of the Montgomery-Åsberg Depression Rating Scale (MADRS, depressive symptoms) at Visit 6 and Visit 8
5. Weekly change from baseline of the patients daily total sleep time (in minutes), sleep onset latency at night (in minutes), recuperation of night sleep (5-point Likert scale, 1 = very much; 5 = not at all), and time awake at night (in minutes), number of nightmares last night (0, 1, 3, 4 or more) and intensity of nightmares (5-point Likert scale, 0 = not at all; 5 = extreme) assessed with sleep diaries during Visit 2 – Visit 8
6. Change from baseline of PTSD symptoms assessed with the PTSD Checklist for DSM-5 (PCL-5) at Visit 6 and Visit 8
7. Change from baseline of the Borderline Symptom List 23 (BSL-23) score at Visit 6 and Visit 8
8. Change from baseline of the Health-Related Quality of Life (EQ-5D) score at Visit 6 and Visit 8
9. Overall patients status measured by the Patient Global Impression of Change (PGIC) at Visit 6 and Visit 8
10. Change from baseline of the Social and Occupational Functioning Assessment Scale (SOFAS) at Visit 6 and Visit 8
11. Change from baseline of the Pittsburgh Sleep Quality Index (PSQI) at Visit 6 and Visit 8
12. Change from baseline of symptoms of PTSD and complex PTSD according to ICD-11 assessed with the International Trauma Questionnaire (ITQ) at Visit 6 and Visit 8
13. Change from baseline of THC withdraw symptoms assessed with the Marijuana Withdrawal Checklist (MWC) at Visit 6, Visit 8, and Visit 9
14. Responder analysis: proportion of patients showing improvement in nightmares (change from baseline) defined as decrease of CAPS-IV B2 ≥50% assessed at the end of treatment (Visit 8)
15. Remitter analysis: proportion of patients showing full remission of nightmares defined as CAPS-IV B2 = 0, assessed at the end of treatment (Visit 8)

### Participant timeline

Every study site will assess patients for eligibility to enroll into the study. Before enrolment, every patient will receive full oral and written information about the nature, purpose, expected advantages and possible risks of the trial. The patient will agree to participate in the trial by signing the informed consent form. Patients, who meet all inclusion criteria, no exclusion criteria and who have given their written informed consent will be randomized for study treatment in one of the two study arms. Eligible patients will be screened up to 10 days prior to randomization. At randomization, patients will be allocated to IMP or placebo. The individual patient will be treated for 10 weeks. The final examination will be performed at visit 8 (week 10). 2 weeks after end-of study a safety follow-up visit will take place. Please see Table 2 for examinations that will be performed at screening and all assessments performed at baseline and the following seven study visits during the trial.

**Table 2:** Outline of trial visits

Assessments	SC	BL Visit 1 W 0	Visit 2 W 1	Visit 3 W 2	Visit 4 W 3	Visit 5 W 4	Visit 6 W 6	Visit 7 W 8	Visit 8 W 10	FU Visit 9 W 12
**Screening and consent**										
Inclusion/exclusion criteria	X									
Informed consent	X									
Physical examination^1^	X								X	
Prior and concomitant medi-cation and non-drug therapy	X	X	X	X	X	X	X	X	X	X
Medical history	X									
IMP dispersion		X	X	X	X	X	X	X		
IMP return			X	X	X	X	X	X	X	
**Safety**										
Blood test^2^	X								X	
Cannabis test	X									
Pregnancy test	X	X		X		X	X	X	X	
ECG	X								X	(X)^3^
Blood pressure, pulse	X	X	X	X	X	X	X	X	X	X
Weight	X	X					X		X	X
Adverse event recording		X	X	X	X	X	X	X	X	X
**Effectiveness**										
**Primary**										
CAPS-IV B2									X	
**Secondary**										
CAPS-IV B2	X	X	X	X	X	X	X	X		
CAPS-5	X^a^	X^b^					X^b^		X^b^	
PSQI-A		X					X		X	
MADRS	X	X				X			X	
Sleep diary		X	X	X	X	X	X	X	X	
PCL-5		X					X		X	
BSL-23		X					X		X	
EQ-5D		X					X		X	
PGIC							X		X	
SOFAS		X					X		X	
PSQI		X					X		X	
ITQ		X					X		X	
MWC		X					X		X	X
**Others**										
Demographics	X									
CTQ	X									
MINI-5	X									
ITI	X									

*SC* Screening, *W.* week ± 3 days, *BL* Baseline and randomization, *FU* Post-trial follow-up, *CAPS-IV B2* B2 score of the Clinician-Administered PTSD Scale for DSM-IV (frequency and intensity of nightmares), *CAPS-5* Clinician-Administered PTSD Scale for DSM-5 (a = last month version, b = last week version), *PSQI-A* Pittsburgh Sleep Quality Index-Addendum for PTSD, *MADRS* Montgomery–Åsberg Depression Rating Scale, *PCL-5* PTSD Checklist for DSM-5, *BSL-23* Borderline Symptom List 23, *EQ-5D* Health-Related Quality of Life, *PGIC* Patient Global Impression of Change, *SOFAS* Social and Occupational Functioning Assessment Scale, *PSQI* Pittsburgh Sleep Quality Index, *ITQ* International trauma questionnaire, *MWC* Marijuana Withdrawal Checklist, *CTQ* Childhood trauma questionnaire, *MINI-5* The Mini International Neuropsychiatric Interview for DSM-5, *ITI* International Trauma Interview

^1^Physical examination includes height and weight of the patient and the skin, abdomen, respiratory system, head and extremities will be examined

^2^blood test s include haematology (erythrocytes, haemoglobin, haematocrit, platelets, leukocytes (including neutrophils, eosinophils, basophils, lymphocytes, monocytes) and serum chemistry (sodium, potassium, chloride, calcium, creatinine, alanine aminotransferase, alkaline phosphatase, gamma glutamyl transferase, aspartate aminotransferase, bilirubin, lipase, thyroid stimulating hormone)

^3^In case of significant abnormal ECG findings at the visit 8 (week 10), a further ECG will be recorded at the follow-up visit

### Rational for selection of doses

Due to the experience from previous trials with cannabis-based medication in healthy individuals and clinical groups, including patients with PTSD, the daily dose was determined to be between 2.5 mg per day to 15 mg per day (single administration) with an increase in dose by 2.5 mg every third/fourth day (see Investigators Brochure, Edition Number Final 4.0, Date of release: 30SEP2020, page 30ff, 6. Effects in Humans). The Investigators Brochure recommends 30 mg dronabinol per day as maximal dose. As single dosing will be applied in this study, the maximal daily dose of dronabinol has been restricted to 15 mg which also corresponds to our clinical experience. Effects of THC on nightmares in PTSD have been found in an open label pilot study with 10 mg per day of THC (5 mg twice a day), starting with 5 mg per day (2.5 mg twice a day) [18]. However, pharmacokinetic parameters of dronabinol show high intra-individual variability. To determine the optimal individual dose with respect to efficacy (decrease of intensity and frequency of nightmares) and side effects, up-titration in steps of 2.5 mg dronabinol per day will be performed with an increase every third/fourth day. Alternating increase every third and every fourth day has been chosen to take into account that the clinical visits will take place every seventh day (once a week) in the up-titration period (first four weeks of treatment, Visit 1 to Visit 5).

### Sample size

The sample size calculation is based on the primary endpoint “Clinician-Administered PTSD Scale-IV (CAPS-IV) B2 score at 10 weeks” in the full analysis set including all randomized patients who received the study medication at least once. The aim is to show a lower average score at 10 weeks in the intervention group (I) than in the control group (C). Sample size calculation will be based on a two-sided, two-sample t-test. Adjustment for baseline scores in an ANCOVA model which is applied for the primary efficacy analysis will increase the power compared with a two-sample t-test, which ignores the influence of different baseline values. Therefore, this strategy for sample size calculation is a conservative procedure. In a random-effects meta-analysis of four RCTs investigating the effect of prazosin on PTSD nightmares, a combined standardized mean difference (Cohen’s d) of 0.5 (95% CI = 0.03–0.96) was observed [1]. We conservatively assume a standardized effect which is a little smaller and given by 0.45. This assumed effect can be considered as clinically relevant because distressing nightmares are an independent risk factor for comorbidity and severity in PTSD patients. Nightmares also contribute to alcohol and substance abuse, suicidal ideation, and even completed suicide [17]. As studies assessing minimal clinically important difference (MCID) for reduction of nightmares in PTSD are currently missing, clinical significance cannot be used to calculate sample size.

The required sample size to detect this effect of 0.45 with a power of 0.8 is 158 (79 per group) calculated with nQuery Version 8.2.1.0. We conservatively assume that there will be no more than 10% of patients who are randomized but never get the study medication. Therefore, the total number of patients to be recruited is 176 (158/176 = 0.9) or 88 per group.

### Recruitment

Patients will be recruited via the in- and outpatients center at the recruiting centers. Flyer, advertising the study, will be distributed at other treatment centers and private practice for psychiatry and psychotherapy.

### Randomization and blinding

Patients will be randomized at baseline in a 1:1 allocation using block randomization with fixed block lengths stratified by the study center. Randomization lists are provided by the cooperating pharmacy (Myonex GmbH). Allocation concealment will be ensured, as Myonex GmbH will release the randomisation code after the patient performed baseline assessment (visit 1). Further, block length is unknown to the entire study team. Treatment is blinded by use of a matching placebo. The Sponsor, study personnel, and participants will be blinded to treatment until the database is locked. The investigator is encouraged to maintain the blind as far as possible. In the case of an emergency, access to the study treatment code for an individual subject will be possible at the trial sites. Emergency envelopes containing information to unblind individual participant will be stored at the trial sites. Every trial patient will receive a patient ID card where the address and telephone number of the responsible investigator in case of emergency and the complete address of the trial site will be indicated. The patient ID card contains information that the patient is participating in a clinical trial involving exposure to BX-1 with dronabinol as the active ingredient or corresponding placebo. The decision about necessity of unblinding lies in the responsibility of the investigator or the authorised person.

Staff of all participating study centers will be trained in the study requirements including application of clinical interviews. Clinical interviews will be performed by trained psychologists (master-degree) or medical doctors (in training or with completed training in psychiatry). Training videos of standardized application of CAPS-5, CAPS-IV B2 Item and MADRS will be rated by assessors of the interviews. Raters with divergent ratings will be trained again. Inter-rater-reliability will be calculated for the primary outcome (CAPS-IV B2 Item) after assessment of 10 videos.

The study sites will make maximum effort to follow the participants for the entire study period. We assume that ≈15% of patients (24 participants) who are randomized and received the study medication at least once will not finish the study as planned (“non-completers”). All randomized participants who are prematurely discontinued from the study drug will be considered “off study drug/on study” and will follow the same schedule of events as those participants who continue study treatment.

### Safety

In general, dronabinol is well tolerated, but the patients` individual maximum dose is limited by adverse reactions. Expected adverse reactions are listed in supplementary table S1. Safety assessments include: Blood pressure and heart rate at every visit; assessment of laboratory parameters (safety laboratory) at screening and end of trial (visit 8 in week 10); physical examination at screening and end of trial; ECG at screening, end of trial and in case of significant abnormal ECG findings at visit 8 (week 10) a further ECG at follow-up (visit 9, week 12). Urinary pregnancy test (women only) at screening, baseline, visit 3 (week 2), visit 5 (week 4), visit 6 (week 6), visit 7 (week 8), and visit 8 (week 10). See also Table 2 for an overview of the safety assessments. Standard procedures for reporting of adverse events will be used. Adverse events will be assessed at every visit starting at visit1.

Further safety information and definitions for adverse event (AE), serious AE and Suspected Unexpected Serious Adverse Reactions (SUSAR) are outlined in the online supplemental material.

### Data management

Data collected on each subject will be captured in the medical files (source data) and are consecutively recorded in the eCRF (secuTrial® software). All electronic data will be stored at the server of the Charité Berlin. All essential documents will be kept in the Investigator Site File which will be stored at the study site in accordance with ICH GCP chapter 8.

### Statistical methods – outcome

#### Primary analysis

The aim is to show that the Clinician-Administered PTSD Scale (CAPS-IV) B2 score for the last week at 10 weeks is lower in the intervention group (I) than the control group (C). An ANCOVA model including the baseline score and adjusted for study center will be applied for primary analysis. The global two-sided significance level will be 0.05.

#### Secondary analyses

Descriptive methods will be used to analyse secondary outcomes, including appropriate summary measures of the empirical distribution as well as 95% confidence intervals and descriptive two-sided p-values. For analyses of secondary endpoints considering the primary score at different follow-up times, the same imputation mechanisms as specified below will be applied. For all other secondary endpoints, no imputation is planned.

### Additional analyses

Additionally, sensitivity of the primary analysis will be analysed in different populations to assess the impact of the analysis population and of the imputation method (comparison to per-protocol population, patients with complete cases). All analyses will be done using validated statistical software.

### Analysis population and missing data

Confirmatory analysis will be conducted based on the full analysis set. The full analysis set consists of all randomized patients which received the study medication at least once. Patients are analysed as randomized. This is the population as close as possible to the intention-to-treat population.

The per-protocol population includes all patients without major protocol violations.

Missing values for the analysis of the primary endpoint will be imputed as follows: If the follow-up assessment at 10 weeks is missing but the patient has at least one follow-up assessment after baseline, last-observation-carried-forward will be used for missing value imputation. If the baseline visit is missing but follow-up assessments are available, the baseline value will be imputed using multiple imputation [21].

### Interim analysis

The initially protocol of the study (version 1.1, see supplements) that has been approved by the authorities did not include an interim analysis. As outlined in the background section, results of a clinical trial with cannabinoid medication in patients with PTSD [4] were published after the approval of the initial protocol (version 1.1, see supplements) and led to the decision to include an interim analysis. The amendment outlined in the study protocol version 2.0 included an interim analysis and was approved by the authorities.

We use the conditional rejection principle for the interim analysis in order to account for the initially unplanned analysis. The overall one-sided alpha will be 0.025 (which is equivalent to the initially planned two-sided alpha of 0.05 but a two-sided calculation is not possible due to the non-binding futility boundary). The interim analysis will be done after 79/176 (44.9%) patients are enrolled (79 is half of the needed analyzable patients (158) since currently there are no non-starters—the initial reason for an increased recruitment number). For the boundaries we chose alpha_1_ equal to 0.0008213 which is according to an alpha spending scheme (O´Brien Fleming) with 2 looks and a non-binding futility alpha_0_ of 0.5 (indicating efficacy in the wrong direction).

If the interim analysis indicates continuation, a sample size recalculation will be done using the previously assumed effect size of d = 0.45 for the second recruiting phase after the interim analysis. If the recalculated sample size exceeds an additional number of 80 patients beyond the initially planned 176 patients, a conditional power calculation for a design with *n* = 256 (176 + 80) will be done. If the conditional power is less than 50%, the trial will also be stopped due to futility.

The statistical analysis plan for the interim analysis is listed in the supplements.

### Formal committee

A Data and Safety Monitoring Board (DSMB) will be established to evaluate the data of the interim analysis and give advice to the sponsor to either ‘continue without change’, ‘study interruption’, ‘protocol change’, or ‘study stop’. The DSMB will consist of three experts in the field including one trial statistician. The DSMB is independent of the study organisers.

## Supplementary Information

**Additional file 1. **

**Additional file 2. **

**Additional file 3. **

**Additional file 4. **

**Additional file 5. **

## Data Availability

Not applicable to this manuscript. After study completion and publication of study results, the data set will be available from the corresponding author upon reasonable request.
