# Supplementary material for: Treating nightmares in posttraumatic stress disorder with dronabinol: study protocol of a multicenter randomized controlled study (THC PTSD-trial)
Source: BMC Psychiatry. 2023 May 5;23:319. doi: 10.1186/s12888-023-04818-5 (PMC10161665; doi:10.1186/s12888-023-04818-5)
Supplement: Supplementary file 2 — Additional file 2. [file 12888_2023_4818_MOESM2_ESM.docx]

**Supplementary Material**

Concomitant pharmacotherapies

The following concomitant therapy that interferes with the treatment evaluation of the Investigational Medicinal Product (IMP) is not permitted:

(1) Cannabis-based medicine

Dronabinol or other cannabis-based medicine is not permitted during the trial or within 3 months prior to screening. The intake of such medication has to be clarified with the patient in an interview. In addition, drug abuse screening test is performed. The investigator may not apply such medication during the course of the trial and has to inform the patient that it is not allowed during the trial.

(2) Cannabis herb

Consumption of cannabis herb/marijuana is not permitted during the trial or within 3 months prior to screening. The consumption has to be clarified with the patient in an interview. In addition, drug screening test is performed at screening. The investigator should inform the patient that the consumption is not allowed during the trial.

(3) Sleep medication

Sleep medication that has been initiated 4 or more weeks before screening can be continued at stable dose during the trial.

Initiation of sleep medication 4 weeks prior screening and during the trial is not allowed. I.e., initiation of all benzodiazepines, non-benzodiazepines and other sleep medication are not allowed 4 weeks prior to screening and during the trial.

As examples the following active ingredients (not trade names) are considered as non-benzodiazepines and other sleep medication. Please note that this list is not extensive:

- All benzodiazepines

- Non-benzodiazepines: zaleplon, zolpidem, zopiclon,

- Other sleep medication: Chloralhydate, Melatonine, Antihistaminergic agents (diphenhydramine, doxylamine, promethazine), mirtazapine, trazodone, trimipramine, doxepin, pipamperon, melperon)

(4) Alpha adrenergic agents (where some evidence of effect on nightmares in PTSD patients has been shown)

Alpha adrenergic agents that has been initiated 4 or more weeks before screening can be continued at stable dose during the trial.

Initiation of alpha adrenergic agents 4 weeks prior screening and during the trial is not allowed.

As examples the following active ingredients (not trade names) are considered as alpha or beta adrenergic agents. Please note that this list is not extensive:

- Doxazosin, prazosin, terazosin, clonidine

Monitoring

The trial site will be monitored by Clinical Trial Office (CTO) Charité to ensure the quality of the data collected. The objectives of the monitoring procedures are to ensure that the trial subject’s safety and rights as a study participant are respected, that accurate, valid and complete data are collected, and that the trial is conducted in accordance with the trial protocol, the principles of GCP and local legislation.

All investigators agree that the monitor regularly visits the trial site and assure that the monitor will receive appropriate support in his activities at the trial site.

The declaration of informed consent includes a statement to the effect that the monitor has the right – while observing the provisions of data protection legislation – to compare the case report forms (eCRFs) with the trial subject’s medical records (doctor’s notes, ECGs, laboratory printouts etc.). The investigator will secure access for the monitor to all necessary documentation for trial-related monitoring. The aims of the monitoring visits are as follows:

- To check the declarations of informed consent.
- To monitor trial subject safety (occurrence and documentation/reporting of AEs and SAEs).
- To check the completeness and accuracy of entries on the eCRF.
- To validate the entries on the eCRF against those in the source documents (source data verification, SDV).
- To evaluate the progress of the trial.
- To evaluate compliance with the trial protocol.
- To assess whether the trial is being performed according to GCP at the trial site.
- To discuss with the investigator aspects of trial conduct and any deficiencies found.

A monitoring visit report is prepared for each visit describing the progress of the clinical trial and any problems (e.g. refusal to give access to documentation).

Safety

Expected adverse reactions of BX-1 are listed in table S1.

**Table S1: Reported adverse reactions of BX-1**

| **System Organ Class** | **Adverse Reactions** | **Frequency** |
| --- | --- | --- |
| Psychiatric disorders | Anxiety/restlessness, depression, euphoria, sleep disorder  Thinking abnormal | Common  Uncommon |
| Nervous system disorders | Dizziness, headache, disturbance in attention, memory impairment, speech difficulties  Tremor, Hypotonia | Common  Uncommon |
| Ear and labyrinth disorders | Vertigo | Common |
| Cardiac disorders | Tachycardia | Uncommon |
| Vascular disorders | Hypertension, | Uncommon |
| Respiratory, thoracic and mediastinal disorders | Dry nose  Dyspnoea | Common  Uncommon |
| General disorders and administration site conditions | Tiredness  Asthenia, feeling abnormal | Very common  Common |
| Gastrointestinal disorders | Nausea, constipation, diarrhoea, abdominal pain upper, dry mouth  Flatulence, vomiting, hypoaesthesia oral | Common  Uncommon |
| Musculoskeletal And Connective Tissue Disorders | Muscular Weakness | Common |
| Metabolism and nutrition disorders | Increased appetite, loss of appetite | Common |
| Skin and subcutaneous tissue disorders | Sweating, local hypersensitivity reactions | Common |
| Eye disorders | Pressure behind the eyes  Dry eye, abnormal sensation in eye | Common  Uncommon |

**Note:** The following frequency categories are used for the evaluation of these adverse reactions: Very common (≥ 1/10), Common (≥ 1/100 to < 1/10), Uncommon (≥ 1/1,000 to < 1/100). Source: investigator brochure (IB) of BX-1, Ed.4.0, 30.Sep 2020; please refer to table 9 of the IB.

Due to the known effects of dronabinol, BX-1 should be administered with caution to patients with mania, depression or schizophrenia, heart diseases, history of seizure-related diseases and history of substance abuse. There might be additional or synergistic effects on central nervous system adverse reactions in patients treated concomitantly with sedative, hypnotic or other psychoactive medicinal products. BX-1 is not intended for the use in pregnant or breastfeeding women.

In order to minimize the risk for patients, patient groups with a significant risk are excluded from trial participation.

In addition to the risks related to the investigational medicinal product, there is a trial procedure related risk. Blood sampling might be painful, or the patients might temporarily become dizzy. Patients may also experience intermittent complaints like re-bleeding or puncture site bruises, blood clot (thrombus) in the punctured vessel (rarely), puncture site infections (rarely) or mechanical nerve damage (very rarely). After initial irritation, vein puncturing for blood sampling is usually painless and hardly noticeable.

The total volume of blood sampling during the entire clinical trial will be approximately 18 ml within approximately 14 weeks and is far below the volume of a normal blood donation. However, in single cases it might be necessary to repeat single blood sampling, e.g. an additional sample has to be taken due to re-measurements of clinical laboratory samples or due to bad vein conditions and a new sample must be taken from another vein, etc. Thus, the total blood volume may deviate in singles cases by a small amount. No safety related risk is expected from this blood sampling.

In case of emergency, standard emergency procedures will be employed. The responsible investigator is to be consulted and informed immediately. He/she will provide all the necessary emergency equipment and specially trained staff to handle emergency events during this clinical trial. All cases of emergency have to be immediately reported as required to the sponsor comprising legal stipulations.

Definitions of AE, SAE, and SUSAR

**Adverse Event (AE)**

An adverse event is any untoward medical occurrence in a patient or clinical trial subject administered a medicinal product and which does not necessarily have a causal relationship with this treatment. An AE could be diseases, signs or symptoms which occur or worsen after enrolment of the patient in the clinical trial.

**Serious Adverse Event (SAE)**

A ‘serious adverse event or serious adverse reaction’ is any untoward medical occurrence or effect that at any dose: results in death, is life-threatening, requires hospitalisation or prolongation of existing hospitalisation, results in persistent or significant disability or incapacity, or is a congenital anomaly or birth defect; other medically important event.

**Adverse Reactions**

Adverse reactions are all untoward and unintended responses to an investigational medicinal product related to any dose administered.

**Suspected Unexpected Serious Adverse Reactions (SUSAR)**

A Suspected Unexpected Serious Adverse Reaction (SUSAR) is any suspected adverse reaction related to the study treatment that is both serious and unexpected. “Unexpected” means that the nature and severity of the adverse reaction are not consistent with the information about the study medication in question set out in the reference safety information. The evaluation of expectedness will be done by the sponsor.

**Treatment of (S)AEs**

All AEs should be treated appropriately. Such treatment may include changes in study drug treatment including possible interruption or discontinuation, starting or stopping concomitant treatments, changes in the frequency or nature of assessments, hospitalization or any other medically required intervention.

**Assessment of SAEs**

As far as possible, each AE should be evaluated to determine: (1) the severity grade (CTCAE v5.0), (2) its relationship to the study drug (assessment of causality), (3) its duration (start and end dates or if continuing at final exam), (4) action taken (no action taken; study drug dosage adjusted/temporarily interrupted; study drug permanently discontinued due to this AE; hospitalization), and (5) whether it constitutes a serious adverse event (SAE).

**Assessment of Seriousness**

Seriousness shall be determined according to the definition above. Furthermore, medical and scientific judgment should be exercised in deciding whether expedited reporting is appropriate in other situations, such as important medical events that may not be immediately life-threatening or result in death or hospitalization but may jeopardize the subject or may require intervention to prevent one of the other outcomes listed in the definition above. These should also usually be considered serious. Examples of such events are intensive treatment in an emergency room, or at home for allergic bronchospasm, blood dyscrasias or convulsions that do not result in hospitalisation, or development of drug addiction or drug abuse.

**Assessment of intensity**

**CTCAE grading:**

Grade 1 Mild; asymptomatic or mild symptoms; clinical or diagnostic observations only; intervention not indicated.

Grade 2 Moderate; minimal, local or noninvasive intervention indicated; limiting age-appropriate instrumental ADL*.

Grade 3 Severe or medically significant but not immediately life-threatening; hospitalization or prolongation of hospitalization indicated; disabling; limiting self care ADL.

Grade 4 Life-threatening consequences; urgent intervention indicated.

Grade 5 Death related to AE.

**Assessment of causality**

Causal relationship between the occurrence of an AE and the administration of the IMP as assessed by the investigator according to the available data:

Related**:** There is a reasonable causal relationship, which means that there is evidence to suggest a causal relationship. The adverse event could medically (pharmacologically/ clinically) be attributed to the investigational product under trial in this protocol.

Not related: There is no reasonable causal relationship, which means that there is no evidence to suggest a causal relationship. The adverse event could not medically (pharmacologically/ clinically) be attributed to the investigational product under trial in this protocol.

**Documentation of AEs and SAEs**

All Serious Adverse Events (SAEs) and all Adverse Events (AEs) need to be documented, no matter if the Investigator suspects a causal connection to the investigational product or not. The documentation needs to include the type of event, start, duration, severity and causality, outcome, action taken.

Related signs, symptoms and laboratory changes should be summarized to a specific disease. The event will be recorded in the CRF SAEs need to be documented on a separate SAE form.

Out of normal range laboratory data need to be analysed concerning their clinical relevance by the Investigator – and if relevant documented as an AE itself. CTCAE criteria will be applied to laboratory values.

All adverse events need to be followed until they subside or stabilize.

The Sponsor will carefully document all SAEs reported by the Investigator. His documentation will be sent to the relevant regulatory authorities and to relevant authorities of other European member states and other contracting states of the EWR agreement, if the study is run in their territory and if they so request.

**Reporting of SAEs**

To ensure patient safety all SAEs occurring after the patient has provided informed consent must be reported, regardless of causality, by the local investigator to the Sponsor within 24 hours of learning its occurrence. The local Investigator is responsible for reporting all adverse events reported by the participant or detected by the study staff without delay and within 24 hours to the Sponsor's designee (CTO Charité, Pharmacovigilance Department). It has to be documented on the appropriate pages of the case report form (CRF).

The Investigator will report any Serious Adverse Event promptly after becoming aware to the Sponsor’s designee (CTO Charité, Pharmacovigilance Department) and will afterwards send an extended written record.

**Reporting of Suspected Unexpected Serious Adverse Reactions (SUSARs)**

The Sponsor’s designee (CTO Charité) will report all suspicious cases of Suspected Unexpected Serious Adverse Reactions (SUSARs) which had been occurred in one of clinical trials conducted by the same sponsor with the same drug substance/IMP to the relevant Ethics Committee, the relevant regulatory authorities and to relevant regulatory authorities of other European member states and other contracting states of the EWR agreement, if the study is run in their territory immediately, at the latest 15 days after it becomes known. He will also inform all Investigators involved in the trial.

In case of a fatal or life threatening SUSAR the Sponsor will report all information relevant for judging the event immediately, at the latest 7 days after the event becomes known to the relevant Ethics Committee, the relevant regulatory authorities and to relevant regulatory authorities of other European member states and other contracting states of the EWR agreement, if the study is run in their territory as well as to all Investigators involved in the trial. After a further 8 days all further relevant information must be available.

**Other safety issues requiring expedited reporting**

The Sponsor’s designee will immediately, at the latest 15 days after it becomes known report all circumstances that require a revision of the risk-benefit analysis to the relevant Ethics Committee, the relevant regulatory authorities and to relevant regulatory authorities of other European member states and other contracting states of the EWR agreement, if the study is run in their territory. This especially includes:

- - Singular cases of expected severe adverse events with an unexpected outcome.
  - Increased incidence of expected severe adverse events that are judged as being clinically relevant.
  - SUSARs which occur after termination of the clinical trial (up to 8 days (five times half-life of 36 h) after termination or exclusion)
  - Events related to study procedures or development of the study medication, which could affect a subject’s safety.

All person-related data will always be transmitted pseudonymised. Before reporting a SUSAR the subject will be unblinded.

**Follow-up of adverse events**

Once an AE is detected, it should be followed until its resolution or stabilisation, and assessment should be made at each visit (or more frequently, if necessary) of any changes in severity, the suspected relationship to the study, the interventions required to treat it and the outcome.

Follow-up information is sent to the same address to which the original SAE Report Form was sent, using a new SAE Report Form stating that this is a follow-up to the previously reported SAE and giving the date of the original report. Each re-occurrence, complication, or progression of the original event should be reported as a follow-up to that event regardless of when it occurs. The follow-up information should describe whether the event has resolved or continues, if and how it was treated, whether the patient continued or withdrew from study participation.

For a follow-up report to the authorities, the investigator may be required to collect further information for a detailed description and a final evaluation of the case, including copies of hospital reports, autopsy reports, or other relevant documents.

**Pregnancy**

The investigator will collect pregnancy information on any female patient, who becomes pregnant while participating in this clinical trial. The investigator will record pregnancy information on the SAE form (under other medically important condition) and submit it to the sponsor immediately (i.e. within 24 hours after acknowledgment of a patient’s pregnancy). The receipt of reports concerning pregnancy will be confirmed immediately, but not later than three (3) working days after the receipt. Without confirmation after three working days, the investigator has to transmit the report again on the following working day.

Any patient who becomes pregnant while participating in the trial should discontinue treatment immediately and will be withdrawn from the trial.

The pregnancy must be followed up to determine outcome (including premature termination) and status of mother and child. Generally, follow-up will be no longer than 3 months following the estimated delivery date. Any follow-up information will be sent to the sponsor immediately (i.e. within 24 hours after acknowledgment).

Newly diagnosed pregnancy in a clinical trial patient is not considered to be an AE or SAE unless meeting seriousness criteria, or it is suspected that the IMP interacted with a contraceptive method and led to pregnancy.

Pregnancy complications and elective terminations for medical reasons must be reported as an AE or SAE. Spontaneous abortions must be reported as an SAE.

Furthermore, any SAE occurring as in association with a pregnancy (in cases where the pregnancy occurred after the end of the trial participation of the patient) brought to the investigator’s attention after the patient has completed the trial and considered by the investigator as reasonably related to the IMP must be promptly reported to the sponsor.

Premature termination of the individual patient

Subjects must be informed about their right to withdraw from the study at any time and for any reason without sanction, penalty, or loss of benefits to which the subject is otherwise entitled. Withdrawal from the study will not jeopardize their future medical care or relationship with the investigator. Subjects will be asked to specify the reason for their termination, but have the right not to answer.

The investigator may decide to withdraw a subject from the study at any time with reasonable rationale. The subject’s future care will not be influenced by a decision, voluntary or otherwise, to withdraw from the study. All reasonable efforts should be made to retain the subject in the study until completion of the study.

The reason for withdrawal of a patient should be recorded, if possible, in the eCRF and in the

subject’s records (Withdrawal of Consent Form).

A subject who withdraws Informed Consent before randomization or who develops a condition infringing with selection criteria before randomization is defined as a screening failure. No follow‐up of screening failures will be performed.

The following events must cause premature termination of the individual subject:

- Unblinding in case of a medical emergency
- The participant meets an exclusion criterion (either newly developed or not previously recognized) that precludes further study
- Pregnancy
- Consent withdrawn by patient
- Events with a score > 3 according to the CTCAE catalogue (Common Terminology Criteria for Adverse Events, Version 5.0)
- Complete missing of two consecutive visits

Premature termination of the clinical study

The following events must cause premature termination of the clinical trial:

• Unjustifiable risk and negative risk-benefit analysis (decision taken by the sponsor)

• New scientific evidence provided during the study that could affect the patient’s safety (benefit-risk analysis no longer positive)

• Decision of the Sponsor or the Competent Authority that the study should be discontinued or because of withdrawal of a positive vote by a responsible ethics committee.

Sponsor may terminate the trial at an individual study centre, if

• the investigator no longer considers the risk-benefit ratio to be reasonable,

• the centre’s recruitment rate is too low,

• the drop-out rate is unusually high, or

• the investigator does not comply with the protocol or other regulations with the necessary care.

**Follow-up and continuing treatment after regular / premature termination**

After the end of the trial, the patients will be treated according to local standard practice. No further post-trial therapy will be offered by the sponsor.

In the case of premature termination, the reason for withdrawal must be entered on the appropriate case report form (CRF) page and must be followed for safety and efficacy until 8 days (five times half-life of 36 h) after discontinuation.

Audits

Authorised representatives of the Sponsor, a regulatory authority, or an Independent Ethics Committee (IEC) may visit the centre to perform audits or inspections, including source data verification. The purpose of a Sponsor audit or inspection is to systematically and independently examine all study related activities and documents to determine whether these activities were conducted, and data were recorded, analysed, and accurately reported according to the protocol, Good Clinical Practice (GCP), guidelines of the International Conference on Harmonisation (ICH), and any applicable regulatory requirements.

Ethics and Dissemination

This trial will be conducted in accordance with the current ICH-GCP-guidelines. Good Clinical Practice (GCP) is an international ethical and scientific quality standard for designing, conducting, recording and reporting trials that involve the participation of human subjects. Compliance with this standard provides public assurance that the rights, safety and well-being of trial subjects are protected, consistent with the principles that have their origin in the Declaration of Helsinki, and that the clinical trial data are credible.

Study protocol, patient information and consent form will be presented to the relevant Ethics Committee for survey. According to AMG § 42 (1) and GCP-V § 7 the study will only start after ethics approval has been granted. The Ethics Committee will immediately be informed (by the Sponsor) of all changes to the protocol (according to GCP-V § 10) and of all events that could affect a patients safety. The Ethics Committee will also be informed of all suspected SUSARs and of regular or premature termination of the study. The results of study will be published at clincialtrial.gov, international peer-revied journals and presented at international conferences.
