# Supplementary material for: Treating nightmares in posttraumatic stress disorder with dronabinol: study protocol of a multicenter randomized controlled study (THC PTSD-trial)
Source: BMC Psychiatry. 2023 May 5;23:319. doi: 10.1186/s12888-023-04818-5 (PMC10161665; doi:10.1186/s12888-023-04818-5)
Supplement: Supplementary file 3 — Additional file 3. [file 12888_2023_4818_MOESM3_ESM.pdf]

Landesamt für Gesundheit und Soziales

Ethik-Kommission des Landes Berlin

Berlin

KKS Charité

29. JAN. 2020

Verteil:

LAGeSo 20 Jahre

Landesamt für Gesundheit und Soziales Berlin
Postfach 310929, 10639 Berlin (Postanschrift)

Koordinierungszentrum für Klinische Studien der Charité
Dr. Uwe Behrens
Augustenburger Platz 1
13353 Berlin

vorab per Fax: 030-450-553 937

Geschäftszeichen (bitte immer angeben)
19/0384 - IV E 11

Dienstgebäude:
Turmstraße 21, 10559 Berlin

Bearbeiter/in:
Urs Schulte
Zimmer: A.06.29

Telefon: 030902291226

Telefax: 03090283383

E-Mailadresse:
urs.schulte@lageso.berlin.de
(nicht für Dokumente mit elektronischer Signatur)

Datum: 22.01.2020

Klinische Prüfung eines Arzneimittels: Antrag auf zustimmende Bewertung einer klinischen Prüfung, § 42 Abs. 1 Arzneimittelgesetz (AMG), § 7 Abs. 1 Verordnung über die Anwendung der Guten Klinischen Praxis bei der Durchführung von klinischen Prüfungen von Arzneimitteln am Menschen (GCP-V).

Antrag vom:	18.10.2019
Eingang am:	22.10.2019
Sponsor:	Charité - Universitätsmedizin Berlin
Bevollmächtigter Vertreter des Sponsors:	Koordinierungszentrum für Klinische Studien der Charité
Leiter der klinischen Prüfung:	Dr. Prof. Dr. Stefan Röpke, 12200 Berlin
EudraCT-Nummer:	2019-002211-25
Prüfplancode:	THC-PTSD-Trial
Titel der klinischen Prüfung:	Treating Nightmares in Posttraumatic Stress Disorder with Dronabinol: A Randomized Controlled Study (THC PTSD-trial)

Nachrichtlich an:

Bundesinstitut f. Arzneimittel u. Medizinprodukte

Sehr geehrte Frau Bölscher,

hiermit bestätige ich den Eingang Ihres Schreibens vom 02.01.2020, eingegangen am 07.01.2020, mit dem Sie nachfolgend genannte Unterlagen eingereicht haben:

Anlage 1	"Durchgeführte Bearbeitungen"
Anlage 2	Protocol Version 1.1 vom 19.12.2019, a) clean, b) tracked changes
Anlage 3	Deutsche Synopse Version 1.1 vom 19.12.2019, a) clean, b) tracked changes
Anlage 4	Patienteninformation Version 1.1 vom 19.12.2019, a) clean, b) tracked changes
Anlage 5	Instruktionen zur Medikamenteneinahme Version 1.1 vom 19.12.2019, a) clean, b) tracked changes
Anlage 6	Begleituntersuchung Sexualfunktion Version 1.1 vom 20.12.2019, a) clean, b) tracked changes
Anlage 7	Begleituntersuchung Nacherhebung Version 1.0 vom 19.12.2019
Anlage 8	Begleituntersuchung virtuelle Realität Version 1.1 vom 03.12.2019
Anlage 9	Versicherungsbestätigung
Anlage 10	Ausdrucke/Dateien des eCRF, Datensatztabelle (Excel) nur auf CD
Anlage 11	Nachreichung für Prüfzentrum Hamburg, Dr. Biedermann: aktualisierte Angaben zur Prüfstelle

Die mit der zustimmenden Bewertung vom 21.11.2019 verbundenen Bedingungen sind damit eingetreten.

Ich danke für die Berücksichtigung der erteilten Hinweise.

Ich bitte Sie um Mitteilung des kalendermäßigen Beginns der o.g. klinischen Prüfung in Deutschland.

Für die Durchführung des o.g. Vorhabens wünsche ich Ihnen viel Erfolg.

Mit freundlichen Grüßen

Bräutigam

Dr. med. Katrin Bräutigam
Stellv. Vorsitzende des Ausschusses 1
der Ethik-Kommission des Landes Berlin

...

Sendebericht

MFP

ECOSYS M2640idw

Firmware-Version 2S5_2000.001.702 2017.12.08

VCN7909083
28/01/2020 13:44
[2S0_1000.002.002] [2S0_1100.001.003] [2S5_7000.001.601]

Auftr.Nr.: 004511 Gesamtzeit: 0°00'10" Seite: 001

Vollendet

Original-: doc00451120200128134324

28/01/2020 12:11 004921143022281 AEKNO-ETHIK S. 03/05

Ärztekammer Nordrhein
Körperschaft des öffentlichen Rechts

Seite 3 von 5
2020017

Anlage 1 zur Eingangsbestätigung

Bitte diese Seite innerhalb von 3 Tagen zurückfaxen:

FAX-Nummer 0211 / 4302-2279

KOSTENÜBERNAHMEERKLÄRUNG

für die Bewertung von Studienunterlagen durch die Ethik-Kommission der Ärztekammer Nordrhein gemäß der gültigen Gebührenordnung

Nr.	Datum/Zeit	Ziel	Zeit	Typ	Ergebnis	Auflösung/ECM
001	28/01/20 13:44	0021143022279	0°00'10"	FAX	OK	200x100 Normal/Ein

ECOSYS®
