# Supplementary material for: Treating nightmares in posttraumatic stress disorder with dronabinol: study protocol of a multicenter randomized controlled study (THC PTSD-trial)
Source: BMC Psychiatry. 2023 May 5;23:319. doi: 10.1186/s12888-023-04818-5 (PMC10161665; doi:10.1186/s12888-023-04818-5)
Supplement: Supplementary file 4 — Additional file 4. [file 12888_2023_4818_MOESM4_ESM.pdf]

Landesamt für Gesundheit und Soziales

Ethik-Kommission des Landes Berlin

Berlin

Landesamt für Gesundheit und Soziales Berlin
Postfach 310929, 10639 Berlin (Postanschrift)

LAGeSo 20 Jahre

Koordinierungszentrum für Klinische Studien der Charité
Dr. Uwe Behrens
Augustenburger Platz 1
13353 Berlin

vorab per Fax: 030-450-553 937

KKS Charité
29. NOV. 2019
Verteiler: BoS

Geschäftszeichen (bitte immer angeben)
19/0384 - EK 11

Dienstgebäude:
Turmstraße 21, 10559 Berlin

Bearbeiter/in:
Urs Schulte
Zimmer: A.06.29

Telefon: 030902291226

Telefax: 03090283383

E-Mailadresse:
urs.schulte@lageso.berlin.de
(nicht für Dokumente mit elektronischer Signatur)

Datum: 21.11.2019

Klinische Prüfung eines Arzneimittels Antrag auf zustimmende Bewertung einer klinischen Prüfung, § 42 Abs. 1 Arzneimittelgesetz (AMG), § 7 Abs. 1 Verordnung über die Anwendung der Guten Klinischen Praxis bei der Durchführung von klinischen Prüfungen von Arzneimitteln am Menschen (GCP-V).

Antrag vom:	18.10.2019
Eingang am:	22.10.2019
Sponsor:	Charité - Universitätsmedizin Berlin
Bevollmächtigter Vertreter des Sponsors:	Koordinierungszentrum für Klinische Studien der Charité
Leiter der klinischen Prüfung:	Dr. Prof. Dr. Stefan Röpke, 12200 Berlin
EudraCT-Nummer:	2019-002211-25
Prüfplancode:	THC-PTSD-Trial
Titel der klinischen Prüfung:	Treating Nightmares in Posttraumatic Stress Disorder with Dronabinol: A Randomized Controlled Study (THC PTSD-trial)

Nachrichtlich an:
- Bundesinstitut f. Arzneimittel u. Medizinprodukte
- Ethik-Kommission der Ärztekammer Hamburg (Fax: 040/ 20 22 99 410)
- Ethik-Kommission II der Universität Heidelberg Universitätsklinikum Mannheim (Fax: 06 21 / 383-9710)

Sehr geehrter Herr Dr. Behrens,

der Ausschuss 1 der Ethik-Kommission des Landes Berlin hat über das o.g. Vorhaben in seiner am durchgeführten Sitzung beraten und erlässt folgende

Bewertungsentscheidung

1. Die vorbezeichnete klinische Prüfung wird mit den unter Ziffer 2 genannten Bedingungen zustimmend bewertet.

2. Bedingungen:

Prüfplan:

Eine Begründung sowohl für die Wahl der Startdosis, von Dauer und Dosisstufen der Titrationsphase als auch der Maximaldosis ist zu ergänzen (justification of dose).

Exclusion Criteria: Laut ICF (S7) gelten auch epileptische Krampfanfälle, relevante Herzerkrankungen, bekannte HIV- und/oder aktive Hepatitis-B- oder Hepatitis-C-Infektion, maligne Erkrankung aktuell und anamnestisch als Kriterien, die gegen eine Studienteilnahme sprechen. Diese sind im Prüfplan zu ergänzen.

3.1 Primary endpoints. Die Definition des primären Endpunkts ist dahingehend zu überarbeiten, dass Aspekte der Auswertung (Adjustierung zu Baseline) zu entfernen sind.
3.2 Secondary endpoints. Die Definition der sekundären Endpunkte ist grundlegend zu überarbeiten. Dabei muss jeder Endpunkt klar definiert werden (inkl. der Zeitpunkte der Erhebung/Auswertung). Widersprüche zur Darstellung der sekundären Endpunkte an anderen Stellen des Prüfplans sind zu korrigieren. Bei Endpunkten des CASP-Instruments muss jeweils die Versionsnummer angegeben werden. Es muss jeweils festgelegt werden, ob absolute Werte für die entsprechenden Zeitpunkte oder Änderungen zu Baseline als Endpunkte definiert sind. Sämtliche Aspekte zur statistischen Analyse der Endpunkte sind hier zu entfernen.

4.1 Inclusion criteria. Die unterschiedlichen Kriterien bzgl. Schwangerschaft und Stillen sind einzeln und eindeutig verständlich darzustellen.

Tabelle 1. Die Darstellung der Zeile „Effectiveness primary / CAPS-IV B2" ist fehlerhaft und ist zu korrigieren. Nur die Erhebung zu W10 ist als primärer Endpunkt definiert. Die restlichen Erhebungen gelten als sekundäre Endpunkte. Zudem ist die Zeile bzgl. „Number of responders and remitters" zu löschen, da hier keine zusätzlichen Daten erhoben werden.
Die Abschnitte „4.3 Subject withdrawal" und „8.1 Premature termination of the individual participant" können zusammengelegt werden. Zudem sind die individuellen Kriterien für den Abbruch der Studienbehandlung aufgrund messbarer Sicherheitsparameter zu spezifizieren und als zwingende auszugestalten, vgl. § 40 Abs. 1 S. 3 Nr. 2 AMG i.V.m. Abschnitt 6.4.6. der ICH Leitlinie E6 (R2) (EMA/CHMP/ICH/135/1995).
4.3 Treatment Discontinuation, S16: Die individuellen Kriterien für den Abbruch der Studienbehandlung sind, soweit sie auf messbaren Sicherheitsparametern beruhen, als zwingende auszugestalten, vgl. § 40 Abs. 1 S. 3 Nr. 2 AMG i.V.m. Abschnitt 6.4.6. der ICH Leitlinie E6 (R2) (EMA/CHMP/ICH/135/1995).

8 Termination, S29: Zwingende kollektive und hinreichend spezifische Sicherheitsparameter für den Abbruch der gesamten Studie fehlen und sind zu ergänzen, vgl. § 40 Abs. 1 S. 3 Nr. 2 AMG. Die Formulierung „may cause" ist daher unangemessen und muss durch „hast to be/must be..." ersetzt werden.

13. Statistical Analysis. Alle Tests auf Unterschiede sollten zweiseitig sein (vgl. ICH Topic E 9 Statistical Principles for Clinical Trials, CPMP/ICH/363/96.) Die Verwendung einseitiger Tests ist zu begründen.

13.1. Sample size estimation. Die Fallzahlschätzung ist dahingehend zu überarbeiten, dass die klinische Relevanz des Behandlungseffekts (bezogen auf den primären Endpunkt) begründet werden muss.
13.3.2. Definition of population for analysis. Die Definition der Intention-to-treat (ITT) Population entspricht nicht dem Prinzip der ICH Topic E 9 Statistical Principles for Clinical Trials, CPMP/ICH/363/96. Die Definition ist entweder entsprechend zu überarbeiten oder die Population ist anders zu bezeichnen (ggf. modified ITT).
13.3.2. Definition of population for analysis. Sämtliche Populationen, die zur Auswertung vorgesehen sind, sind hier zu definieren (s. 13.3.3. „patients with complete cases").
13.3.2. Es muss erläutert werden, ob und für welche Endpunkte, welche Imputationsmethoden verwendet werden. Ist für sämtliche Endpunkte zu sämtlichen Zeitpunkten eine Ersetzung fehlender Werte geplant? Die entsprechenden Methoden müssen beschrieben und begründet werden.
13.3.3. Die Darstellung in den beiden Synopsen (deutsch und englisch) bzgl. lokaler Signifikanzniveaus adjustiert nach Pocock ist konsistent und nachvollziehbar im Kapitel 13. darzustellen.
13.3.3. Es muss im Prüfplan eine detaillierte Beschreibung der ausgewählten Imputationsmethode (inkl. der Festlegung der Imputationsmodelle/-variablen) erfolgen. Zudem muss eine Begründung für die Eignung der anzuwendenden Methoden gegeben werden. Auch die geplanten Sensitivitätsanalysen sind im Voraus festzulegen. (vgl. EMA/CPMP/EWP/1776/99 Rev. 1)
13.3.3. Die Auswertung der sekundären Endpunkte sollte analog der Auswertungsstrategie des primären Endpunkts (ggf. ohne Imputation fehlender Werte) erfolgen (jedoch mit explorativer Interpretation).
Die Erhebung der Endpunkte sollte auch in den letzten 2 Wochen der Studie (nach letzter Gabe des Prüfprodukts) erfolgen.

Patienteninformation:

Es wurden folgende Mängel festgestellt, die zu beheben sind:

- S. 2: Die Ethikkommission erteilt eine zustimmende Bewertung (nicht "Stellungnahme").

- S. 3: Die bei Widerruf der Einwilligung erbetene Teilnahme an einer abschließenden Untersuchung sollte als freiwillig dargestellt werden.

- Instruktionen zur Medikamenteneinnahme, S4/5: Im Text wird von „Tropfen" des Studienmedikaments gesprochen, im Schema dagegen von „Hüben". Zur besseren Nachvollziehbarkeit sollten die Angaben einheitlich sein oder durchweg beide Bezeichnungen verwendet werden. Neben den Hüben/Tropfen pro Tag ist für die Patient*innen auch eine mg-Angabe zu ergänzen.

- S. 10: Es sollte auch dargestellt werden, dass Patienten im Placeboarm keinen klinischen Nutzen erwarten können.

- S.11: Für sämtliche Begleituntersuchungen sollte explizit klargestellt werden, dass sie optional sind und die Hauptstudie auch ohne diese durchlaufen werden kann.

- Es sollte bei Beschreibung der klinischen Studie die Rolle des "finanziellen Förderers" (vgl. Prüfzentrums-Vertrag) Bionorica kurz dargestellt werden, der auf Seite 15 als "das pharmazeutische Unternehmen Bionorica" und Empfänger anonymisierter Daten erscheint, sowie die Rolle der ebenfalls auf Seite 15 erwähnten Canopy Growth Corporation.

- Laut Prüfplan S. 16 ist die Anwendung einer hocheffektiven Verhütungsmethode auch für teilnehmende Männer vorgesehen während der Studie bis 30d nach letzter IMP-Einnahme; diese Information ist im ICF zu ergänzen.

- S. 18: Bei der Erwähnung von ClinicalTrials.gov bzw. www.clinicaltrialsregister.eu sollte

zusätzlich ein geeigneter Suchbegriff, etwa Prüfplancode/Studien-ID des Sponsors, angegeben werden, anhand dessen der Patient die Studie in der Datenbank finden kann.

- S. 19 (Einwilligungserklärung): Neben der Aushändung eines Exemplars der Patienteninformation sollte auch die Aushändigung von Versicherungsbedingungen und Versicherungsschein oder -bestätigung aufgenommen werden.

Deutsche Synopse:
Die deutsche Synopse ist grundlegend zu überarbeiten und in verständlicher und korrekter Sprache einzureichen.

CRF/Erhebungsbögen
CRF/Erhebungsbögen sollten vorgelegt werden.

- S. 12: Es ist die Anschrift des Versicherers anzugeben.

Versicherung:

- Die abgeschlossene, für mehrere Studien geltende Jahresversicherung entspricht nicht den gesetzlichen Anforderungen nach einer Versicherung, deren Umfang auf Basis der Risikoabschätzung für eine konkrete beantragte Studie kalkuliert wurde. Die Möglichkeit, dass Versicherungsfälle anderer Studien die für diese Studie kalkulierte Deckungssumme schmälern können, ist gesetzwidrig. Das gleiche gilt, wenn einzelne Versicherungsleistungen für Teilnehmer dieser Studie aufgrund von Versicherungsfällen anderer Studien gekürzt werden. Die Deckungssumme hat für Versicherungsfälle dieser Studie zur Verfügung zu stehen. Vor diesem Hintergrund ist eine Alternative zu wählen, die diese Deckungssumme und damit die möglichen Versicherungsleistungen für Teilnehmer dieser Studie gewährleistet, etwa eine Garantieerklärung, eine zum Zweck der Sicherung der Summe und der möglichen Leistungen abgeschlossene Rückversicherung oder eine Umstellung der Jahresversicherung auf eine Einzelversicherung. Die Teilnehmer der Studie sind in der Patienteninformation über die gewählte Alternative aufzuklären.

Mit der o.g. Studie darf erst begonnen werden, wenn die vorstehenden Bedingungen eingetreten sind. Hierzu ist eine schriftliche Stellungnahme der Ethik-Kommission einzuholen.

3. Die zustimmende Bewertung erstreckt sich auf die in der Anlage aufgeführten Prüfer, Stellvertreter und Prüfstellen.

4. Es werden nachstehende Hinweise erteilt:

   Prüfplan:

   Layout und Formatierung sind zu überarbeiten (z.B. fehlende Seitenzahl, fehlerhafte Überschrift).
   4.1 Inclusion criteria. Die letzten 4 Kriterien sollen (entsprechend umformuliert) als Ausschusskriterien unter 4.2 definiert werden.

Patienteninformation:

S.8: "alkoholische Getränke dürfen während der Studienteilnahme nicht konsumiert wserden "
- bitte orthographisch korrigieren

Zusatzinformationsblatt zur computergestützen Begleituntersuchung zu Sexualfunktion
Hier sollte etwas klarer und anschaulicher dargestellt werden worin die Begleituntersuchung besteht.

Rechtliche Bewertung

Dem Antrag war stattzugeben und die klinische Prüfung zustimmend zu bewerten. Es wird festgestellt, dass Versagungsgründe gemäß § 42 Abs. 1 Satz 7 AMG nicht vorliegen, sofern die vorstehenden Bedingungen eintreten. Die Zulässigkeit der Bedingung ergibt sich aus § 1 Abs. 1 des Gesetzes über das Verfahren der Berliner Verwaltung in Verbindung mit § 36 Abs. 1 des Verwaltungsverfahrensgesetzes. Die Bedingung ist gegenüber einer Versagung das mildere Mittel. Sie soll sicherstellen, dass die gesetzlichen Voraussetzungen einer zustimmenden Bewertung erfüllt werden.

Unter Berücksichtigung der vorstehenden Bedingungen sind folgende Gründe für die zustimmende Bewertung maßgebend:

- Die Unterlagen und Angaben sind gem. § 7 Abs. 2, Abs. 3 und Abs. 3a GCP-V vollständig eingereicht worden, vgl. § 42 Abs. 1 Nr. 1 AMG.

- Die Unterlagen, einschließlich des Prüfplans, der Prüferinformation und der Modalitäten für die Auswahl der Prüfungsteilnehmer entsprechen dem Stand der wissenschaftlichen Erkenntnisse, insbesondere ist die klinische Prüfung geeignet, den Nachweis der Unbedenklichkeit oder Wirksamkeit eines Arzneimittels, einschließlich der Unterschiede zwischen Männern und Frauen, zu erbringen, vgl. § 42 Abs. 1 Nr. 2 AMG.

- Die vorhersehbaren Risiken und Nachteile der klinischen Prüfung sind gegenüber dem Nutzen für die Person, bei der sie durchgeführt werden soll (betroffene Person), und der voraussichtlichen Bedeutung des Arzneimittels für die Heilkunde ärztlich vertretbar, vgl. §§ 42 Abs. 1 Nr. 3 i.V.m. 40 Abs. 1 Nr. 2 AMG.

- Die betroffenen Personen sind volljährig und in der Lage, Wesen, Bedeutung und Tragweite der klinischen Prüfung zu erkennen und ihren Willen hiernach auszurichten, vgl. §§ 42 Abs. 1 Nr. 3 i.V.m. 40 Abs. 1 Nr. 3 a) AMG.

- Die betroffenen Personen werden nach § 40 Abs. 2 S. 1 AMG aufgeklärt und willigen schriftlich ein, an der klinischen Prüfung teilzunehmen, vgl. §§ 42 Abs. 1 Nr. 3 i.V.m. 40 Abs. 1 Nr. 3b) AMG.

- Die betroffenen Personen werden nach § 40 Abs. 2a S. 1 und 2 AMG informiert und willigen schriftlich ein; die Einwilligung bezieht sich ausdrücklich auch auf die Erhebung und Verarbeitung von Angaben über die Gesundheit, vgl. §§ 42 Abs. 1 Nr. 3 i.V.m. 40 Abs. 1 Nr. 3 c) AMG.

- Es werden keine auf behördliche oder gerichtliche Anordnung in einer Anstalt untergebrachten Personen in die klinische Prüfung einbezogen, vgl. §§ 42 Abs. 1 Nr. 3 i.V.m. 40 Abs. 1 Nr. 4 AMG.

- Die klinische Prüfung soll in einer geeigneten Einrichtung von einem angemessen qualifizierten Prüfer verantwortlich durchgeführt werden und die Leitung wird von einem Prüfer oder Leiter

der klinischen Prüfung wahrgenommen, der eine mindestens zweijährige Erfahrung in der klinischen Prüfung von Arzneimitteln nachweisen kann, vgl. § 42 Abs. 1 S. 7 Nr. 3 i.V.m. § 40 Abs. 1 Nr. 5 AMG.

- Der Prüfer hat sachgerechte Kriterien zur Auswahl angemessen qualifizierter Mitglieder der Prüfgruppe festgelegt und ist geeignet und in der Lage, diese anzuleiten und zu überwachen, vgl. § 42 Abs. 1 S. 7 Nr. 3 i.V.m. § 40 Abs. 1a S. 1 und S. 2 AMG.

- Für jeden Prüfer steht mindestens ein vergleichbar qualifizierter Stellvertreter zur Verfügung, vgl. § 42 Abs. 1 S. 7 Nr. 3 i.V.m. § 40 Abs. 1a S. 3 AMG.

- Eine dem Stand der wissenschaftlichen Erkenntnisse entsprechende pharmakologisch-toxikologische Prüfung wurde durchgeführt, vgl. §§ 42 Abs. 1 Nr. 3 i.V.m. 40 Abs. 1 Nr. 6 AMG.

- Jeder Prüfer wird durch einen für die pharmakologisch-toxikologische Prüfung verantwortlichen Wissenschaftler über deren Ergebnisse und die voraussichtlich mit der klinischen Prüfung verbundenen Risiken informiert, vgl. §§ 42 Abs. 1 Nr. 3 i.V.m. § 40 Abs. 1 Nr. 7 AMG.

- Es liegt eine Versicherung der Studienteilnehmer mit einem angemessenem Umfang vor, §§ 42 Abs. 1 Nr. 3 i.V.m. 40 Abs. 1 Nr. 8, Abs. 3 AMG.

- Für die medizinische Versorgung der betroffenen Person ist ein Arzt verantwortlich, vgl. §§ 42 Abs. 1 Nr. 3 i.V.m. 40 Abs. 1 Nr. 9 AMG.

- Die Anwendung des zu prüfenden Arzneimittels ist nach den Erkenntnissen der medizinischen Wissenschaft angezeigt, um das Leben dieser Person zu retten, ihre Gesundheit wiederherzustellen oder ihr Leiden zu erleichtern, §§ 42 Abs. 1 Nr. 3 i.V.m. 41 Abs. 1 Nr. 1 AMG und/oder

- Die klinische Prüfung ist für die Gruppe der Patienten, die unter derselben Krankheit leiden, wie diese Person, mit einem direkten Nutzen verbunden, §§ 42 Abs. 1 Nr. 3 i.V.m. 41 Abs. 1 Nr. 2 AMG.

<u>Hinweise / Besondere Pflichten</u>
Der Sponsor, der Prüfer und alle weiteren an der klinischen Prüfung beteiligten Personen haben bei der Durchführung der klinischen Prüfung eines Arzneimittels bei Menschen die Anforderungen der guten klinischen Praxis nach Maßgabe des Artikels 1 Abs. 3 der Richtlinie 2001/20/EG des Europäischen Parlaments und des Rates vom 4. April 2001 zur Angleichung der Rechts- und Verwaltungsvorschriften der Mitgliedstaaten über die Anwendung der Guten Klinischen Praxis bei der Durchführung von klinischen Prüfungen mit Humanarzneimitteln (ABl. EG Nr. L 121 S. 34) einzuhalten.

Auf die in §§ 12, 13 der Verordnung über die Anwendung der Guten Klinischen Praxis bei der Durchführung von klinischen Prüfungen mit Arzneimitteln zur Anwendung am Menschen (GCP-Verordnung) vom 09.08.2004 (BGBl. 2004, Teil I, S. 2081, 2086) niedergelegten Anzeige-, Dokumentations- und Mitteilungspflichten wird hingewiesen.Berichte über schwerwiegende unerwünschte Ereignisse oder Verdachtsfälle unerwarteter schwerwiegender Nebenwirkungen (SUSARs) können an die Ethik-Kommission gerne per E-Mail (Susar-EK-Berlin@lageso.berlin.de) übersandt werden.

Die Ethik-Kommission des Landes Berlin geht davon aus, dass alle Prüfer und ihre Stellvertreter vom Sponsor, seinem Vertreter oder Beauftragten hinsichtlich ihrer sich aus dem 2. Gesetz zur Änderung arzneimittelrechtlicher und anderer Vorschriften ergebenden Pflichten v. 19.10.2012 (BGBl. I S. 2192) zur Auswahl, Anleitung, Überwachung und Information der Mitglieder der Prüfgruppe unterrichtet werden, sofern diese Kenntnisse nicht durch Vorlage eines aktuellen Schulungsnachweises bereits belegt sind.

...

Die Ethik-Kommission des Landes Berlin bittet um die vierteljährliche Vorlage einer Liste aller während der o.g. klinischen Prüfung gemeldeten Verdachtsfälle schwerwiegender unerwarteter Nebenwirkungen, beginnend ab dem Zeitpunkt des Beginns der o.g. klinischen Prüfung in Deutschland.
Die Verpflichtungen des Sponsors zur Vorlage von Einzelmitteilungen gem. § 13 Abs. 2 bis 4 GCP-V und eines jährlichen Sicherheitsberichts gem. § 13 Abs. 6 GCP-V bleiben hiervon unberührt.

Unter Hinweis auf § 40 Abs. 1 S. 1 AMG i.V.m. Art. 8 Abs. 3 der Richtlinie 2005/28/EG i.V.m. Abschnitt 7.1 der ICH/GCP-Leitlinie Topic E6 bittet die Ethik-Kommission des Landes Berlin um regelmäßige Vorlage der jährlich zu aktualisierenden Prüferinformationen. Sofern es sich um substantielle Änderungen handelt, ist zugleich ein Antrag auf zustimmende Bewertung einer nachträglichen Änderung gem. § 10 Abs. 1 GCP-V zu stellen.

Die Ethik-Kommission behält sich vor, die vorstehende zustimmende Bewertung des o.g. Antrags zum Schutz der Rechte, der Sicherheit und des Wohlergehens der Prüfungsteilnehmer ganz oder teilweise mit Wirkung für die Zukunft zu widerrufen, wenn sie auf Grund nachträglich eingetretener Tatsachen berechtigt wäre, die zustimmende Bewertung zu versagen.

Die Ethik-Kommission des Landes Berlin vertritt die Auffassung, dass die Verarbeitung der Initialen und/oder des vollständigen Geburtsdatums eines Prüfungsteilnehmers mangels „Ersetzung der identifizierenden Angaben" keine Pseudonymisierung der Gesundheitsdaten i.S.d. § 40 Abs. 2a S. 2 Nr. 1 b)-d) AMG i.V.m. Art. 4 Nr. 5 DSGVO darstellt und daher rechtswidrig ist.

Die Festsetzung und Erhebung der Gebühren erfolgt durch gesonderten Bescheid.

Rechtsbehelfsbelehrung
Gegen diesen Bescheid ist die Klage vor dem Verwaltungsgericht zulässig. Die Klage ist innerhalb eines Monats nach Zugang dieses Bescheides bei dem Verwaltungsgericht Berlin, Kirchstr. 7, 10557 Berlin (Tiergarten), schriftlich oder zur Niederschrift bei dem Urkundsbeamten der Geschäftsstelle oder in elektronischer Form mit einer qualifizierten Signatur im Sinne des Signaturgesetzes versehen (vgl. hierzu www.berlin.de/erv) einzulegen; der Klageschrift soll eine Abschrift beigefügt werden. Die Klage ist gegen das Land Berlin, vertreten durch das Landesamt für Gesundheit und Soziales Berlin, zu richten. Es wird darauf hingewiesen, dass bei schriftlicher Klageeinlegung die Klagefrist nur dann gewahrt ist, wenn die Klage innerhalb dieser Frist bei dem Verwaltungsgericht eingegangen ist.

Mit freundlichen Grüßen
gezeichnet:

Prof. Dr. med. K. Thomas Moesta
Vorsitzender des Ausschusses 1
der Ethik-Kommission des Landes Berlin

ausgefertigt: i. A.

Schulte

Anlagen:

...

An der Beschlussfassung waren folgende Mitglieder beteiligt:

Herr Prof. Dr. med. K. Thomas Moesta (Vorsitzender)	Facharzt für Chirurgie
Frau Dr. med. Juliane Bolbrinker	Fachärztin für Pharmakologie
Frau Dr. med. Britta Oldörp	Fachärztin für Anästhesiologie
Herr Dr. iur. Heiko Krüger	Volljurist, Rechtsanwalt
Frau Dr. rer. medic. Stephanie Roll	Biometrikerin
Herr Dr. rer. nat. Jochen Kotwas	Apotheker
Herr Thomas Friedl	Laie
Herr David Schumann	Laie

Bewertete Unterlagen:

00a	Checkliste (Ethikkommission)
Anlage 01	Bestätigungsschreiben EudraCT Nummer vom 16.05.2019
Anlage 02	THC-PTSD Protokoll, Version 1.0, vom 15.10.2019
Anlage 03	THC-PTSD-Studie, Patienteninformation und Einwilligungserklärung, Version 1.0 vom 15.10.2019
Anlage 04	Modul 2
Anlage 05	(a) Clinical Trial Application Form (modul-1), pdf (Stand: 16.10.2019)<br>(b) -Clinical Trial Application Form (modul-1), XML (Stand: 16.10.2019)
Anlage 06	Prüferinformation: 20190927_BX-1_IB_v 3.0_final_clean_signed
Anlage 07	Synopse, Version 1.0, vom 15.10.2019
Anlage 08	(a) Versicherungsbestätigung<br>(b) Versicherungsbedingungen
Anlage 09	Unterlagen Prüfstelle Charite, CBF (Prüfer: Prof. Röpke)
Anlage 10	Unterlagen Prüfstelle Alexianer St. Hedwig Krankenhaus (Prüferin: Dr. Nikola Schoofs)
Anlage 11	Unterlagen Prüfstelle UKE (Hamburg) (Prüferin: PD Dr. Sarah Biedermann)
Anlage 12	Unterlagen Prüfstelle Mannheim (ZI) (Prüfer: Prof. Dr. Christian Schmahl)
Anlage 13	Sponsor-Prüfzentrumsvertrag (Entwurf)
Anlage 14	(a) Vollmacht (Sponsordelegation)<br>(b) Vollmacht für KKS (Studieneinreichung)
Anlage 15	Liste der Prüfstelle und der Ethikkommissionen (Stand: 16.10.2019)
Anlage 16	THC_Instruktionen_zur_Medikamenteneinahme_V1.0_vom_15.10.2019
Anlage 17	THC Patientenausweis, V1.0, vom 15.10.2019
Anlage 18	THC_Begleituntersuchung im Schlaflabor_V1.0_vom_15.10.2019
Anlage 19	THC_Begleituntersuchung in virtueller Realität_V1.0_vom_15.10.2019
Anlage 20	THC_Begleituntersuchung Sexualfunktion_V1.0_15.10.2019
Anlage 21	THC_Begleituntersuchung visuelle Wahrnehmung_V1.0_vom_15.10.2019docx
Anlage 22	THC_Begleituntersuchung_CANTAB Kognitive Funktion_V1.0_vom_15.10.2019
Anlage 23	THC_Begleituntersuchung_Fahrtauglichkeit_V1.0_vom_15.10.2019
Anlage 24	THC_Begleituntersuchung_Zelluläre Funktion_V1.0_vom_15.10.2019
Anlage 25	Flyer THC PTSD Studie_20190930 -jb

01_GCP-Aufbaukurs-Prof_Otte_Charite.pdf

02_GCP-Aufbaukurs_Tobias_Lotter_Mannheim.pdf

Liste der positiv bewerteten Prüfstellen:

1
Prof. Dr. Stefan Röpke (Prüfer)
Stellvertreter: Prof. Dr. Christian Otte
Charite - Universitätsmedizin Berlin
Campus Benjamin Franklin,
Klinik für Psychiatrie und Psychotherapie
Hindenburgdamm 30, 12200 Berlin

2
Dr. Nikola Schoofs (Prüferin)
Stellvertreter: Felix Müllenmeister
Alexianer St. Hedwig Krankenhaus
Traumaambulanz Berlin
Große Hamburger Str. 5-11, 10115 Berlin

4
Prof. Dr. Christian Schmahl (Prüfer)
Stellvertreter: Tobias Lotter
Zentralinstitut für Seelische Gesundheit (ZI)
Institut für Psychiatrische und Psychosomatische Psychotherapie
J5, 68159 Mannheim

Folgende Prüfstelle konnte wegen der ausstehenden Stellungnahme der beteiligten Ethik-Kommission noch nicht bewertet werden:

3
PD Dr. Sarah Biedermann (Prüferin)
Stellvertreter: Dr. Felix Oberhauser
Universitätsklinikum Hamburg-Eppendorf
Klinik und Poliklinik für Psychiatrie und Psychotherapie
Martinistraße 52, Gebäude West 37, 20246 Hamburg
