# Supplementary material for: Treating nightmares in posttraumatic stress disorder with dronabinol: study protocol of a multicenter randomized controlled study (THC PTSD-trial)
Source: BMC Psychiatry. 2023 May 5;23:319. doi: 10.1186/s12888-023-04818-5 (PMC10161665; doi:10.1186/s12888-023-04818-5)
Supplement: Supplementary file 5 — Additional file 5. [file 12888_2023_4818_MOESM5_ESM.pdf]

Treating Nightmares in Posttraumatic Stress Disorder with Dronabinol: A Randomized Controlled Study (THC PTSD-trial)

THC-PTSD

Statistical Analysis Plan for interim analysis

Version 0.3 of March 3rd 2023

Investigational medicinal product:	Dronabinol
Comparator	Oily Placebo Solution (cannabis aroma)
Indication:	Posttraumatic Stress Disorder
Sponsor:	Charité – Universitätsmedizin Berlin
Financial support:	Bionorica SE
Protocol registry identification:	THC-PTSD
Development phase:	Phase II
EudraCT-Number:	2019-002211-25
Protocol version	Version 2.0 / 01.08.2022

Approved by

Prof. Dr. Stefan Röpke, Principal Investigator

Stefan Roepke

Digital unterschrieben von Stefan Roepke Datum: 2023.03.21 16:12:16 +01'00'

Place and date Signature

Robert Röhle, Statistician

Robert Roehle

Digital unterschrieben von Robert Roehle Datum: 2023.03.23 17:11:36 +01'00'

Place and date Signature

Version	Relevant changes from previous version
0.1	Initial draft
0.2	Addition of demography and baseline variables, secondary endpoints for interim analysis, adverse events, correction of trial visits
0.3	Inclusion of imputation method for primary endpoint<br>Addition of sensitivity analysis for the primary endpoint

TABLE OF CONTENTS

ABBREVIATIONS **3**
1 PURPOSE OF THIS DOCUMENT **4**
2 BACKGROUND **4**
2.1 TRIAL OBJECTIVE 4
2.2 TRIAL DESIGN 4
2.2.1 Study plan *4*
2.2.2 Treatments *4*
2.2.3 Blinding *4*
2.2.4 Inclusion criteria *6*
2.2.5 Exclusion criteria *6*
2.2.6 Randomization *7*
2.2.7 Sample size *7*
3 ANALYSIS SETS **7**
3.1 DEFINITIONS 7
3.2 APPLICATION 7
4 TRIAL CENTRES **7**
5 ANALYSIS VARIABLES FOR THE INTERIM ANALYSIS **7**
5.1 DEMOGRAPHY AND BASELINE CHARACTERISTICS 7
5.2 PRIMARY VARIABLE 8
5.3 SECONDARY VARIABLES 8
6 HANDLING OF MISSING VALUES AND OUTLIERS **8**
6.1 MISSING VALUES 8
6.2 OUTLIERS 8
7 STATISTICAL ANALYSES / METHODS **8**
7.1 GENERAL REMARKS 8
7.2 DEMOGRAPHY AND BASELINE CHARACTERISTICS 8
7.3 PRIMARY ANALYSIS 8
7.4 SECONDARY ANALYSES 9
8 SOFTWARE **9**
9 REFERENCES **9**

Abbreviations

Abbreviation	Term
SAP	Statistical Analysis plan
PTSD	Posttraumatic stress disorder
CAPS	Clinician-Administered PTSD Scale
MADRS	Montgomery-Åsberg Depression Rating Scale
MCID	Minimal clinically important difference
ICH	International Council for Harmonisation of Technical Requirements for Pharmaceuticals for Human Use
AE	Adverse event
SAE	Serious adverse event

1 Purpose of this document

In this document, the unplanned interim analysis for the THC-PTSD trial is described. The Statistical analysis plan (SAP) for the final analysis will be provided in a different document.

2 Background

2.1 Trial objective

Primary objective of the study is to examine the efficacy of oral dronabinol over placebo in reducing nightmares in patients with posttraumatic stress disorder.
Secondary objectives of the study are to examine the efficacy of oral dronabinol in reducing disorder-specific symptoms in patients with posttraumatic stress disorder, and to examine the efficacy of oral dronabinol in reducing sleep disturbance in patients with posttraumatic stress disorder.

2.2 Trial design

The study is a multi-centric, double blind, randomized, placebo-controlled, parallel group interventional exploratory phase II trial.

2.2.1 Study plan

The study plan including all visits is shown in Table 1.

2.2.2 Treatments

Description of study medication / investigational medicinal product
BX-1 is an oily solution containing 25 mg/ml dronabinol. Dronabinol ((-)trans-Δ9-Tetra-hydrocannabinol, Δ9-THC) is the major psychoactive constituent of Cannabis sativa L.. Dronabinol is a clear to amber resin. It is highly lipid soluble. Dronabinol is thermolabile, photolabile and susceptible to oxidation.

Placebo / reference medication
There is no discernible difference between BX-1 and the corresponding placebo with regard to colour, taste, and appearance, thus ensuring the trials double-blind character. However, batch-specific variations of odour and colour may occur. In addition, all medications for the double-blind period will be labelled with a common batch number.
Trial treatments are double-blinded. The medication list and the randomisation list are stored at the pharmacy (St-Hubertus Apotheke) and the allocation to treatment groups will be managed by the pharmacy (St-Hubertus Apotheke) and will not be known to the investigator or any other person involved in the conduct of the trial until completion of the trial, except in case of an emergency (see 5.5).

2.2.3 Blinding

Treatment is blinded by use of a matching placebo. The Sponsor, study personnel, and subjects will be blinded to treatment until the database is locked. Emergency envelopes will be provided by the pharmacy and will be stored in the trial sites. These emergency envelopes could then be used by investigators in case of emergencies.

Table 1: Outline of trial visits

**Assessments**	**SC**	**BL Visit 1 W 0**	**Visit 2 W 1**	**Visit 3 W 2**	**Visit 4 W 3**	**Visit 5 W 4**	**Visit 6 W 6**	**Visit 7 W 8**	**Visit 8 W 10**	**FU Visit 9 W 12**
**Screening and consent**										
Inclusion/exclusion criteria	X									
Informed consent	X									
Physical examination[1]	X								X	
Prior and concomitant medication and non-drug therapy	X	X	X	X	X	X	X	X	X	X
Medical history	X									
IMP dispersion		X	X	X	X	X	X	X		
IMP return			X	X	X	X	X	X	X	
**Safety**										
Blood test[2]	X								X	
Cannabis test	X									
Pregnancy test	X	X		X		X	X	X	X	
ECG	X								X	(X)[3]
Blood pressure, pulse	X	X	X	X	X	X	X	X	X	X
Weight	X	X					X		X	X
Adverse event recording		X	X	X	X	X	X	X	X	X
**Effectiveness**										
**Primary**										
CAPS-IV B2									X	
**Secondary**										
CAPS-IV B2	X	X	X	X	X	X	X	X		
CAPS-5	X[a]	X[b]					X[b]		X[b]	
PSQI-A		X					X		X	
MADRS	X	X				X			X	
Sleep diary		X	X	X	X	X	X	X	X	
PCL-5		X					X		X	
BSL-23		X					X		X	
EQ-5D		X					X		X	
PGIC							X		X	
SOFAS		X					X		X	
PSQI		X					X		X	
ITQ		X					X		X	
MWC		X					X		X	X
**Others**										
Demographics	X									
CTQ	X									
MINI-5	X									
ITI	X									

Note: SC = screening, W. = week +/- 3 days, BL = baseline and randomization, FU = post-trial follow-up, CAPS-IV B2 = B2 score of the Clinician-Administered PTSD Scale for DSM-IV (frequency and intensity of nightmares), CAPS-5 = Clinician-Administered PTSD Scale for DSM-5 (a = last month version, b = last week version), PSQI-A = Pittsburgh Sleep Quality Index-Addendum for PTSD, MADRS = Montgomery–Åsberg Depression Rating Scale, PCL-5 = PTSD Checklist for DSM-5, BSL-23 = Borderline Symptom List 23, EQ-5D = Health-Related Quality of Life, PGIC = Patient Global Impression of Change, SOFAS = Social and Occupational Functioning Assessment Scale, PSQI = Pittsburgh Sleep Quality Index, ITQ = international trauma questionnaire, MWC = Marijuana Withdrawal Checklist, CTQ = Childhood trauma questionnaire, MINI-5 = The Mini International Neuropsychiatric Interview for DSM-5, ITI = International Trauma Interview. 1 Physical examination includes height and weight of the patient and the skin, abdomen, respiratory system, head and extremities will be examined, 2: blood test s include haematology (erythrocytes, haemoglobin, haematocrit, platelets, leukocytes (including neutrophils, eosinophils, basophils, lymphocytes, monocytes) and serum chemistry (sodium, potassium, chloride, calcium, creatinine, alanine aminotransferase, alkaline phosphatase, gamma glutamyl transferase, aspartate aminotransferase, bilirubin, lipase, thyroid stimulating hormone), 3. In case of significant abnormal ECG findings at the visit 8 (week 10), a further ECG will be recorded at the follow-up visit.

2.2.4 Inclusion criteria

- Diagnosis of posttraumatic stress disorder (PTSD) according to DSM-5 with a 20 item CAPS-5 total score ≥ 26
- At least two nightmares a week, an intensity score ≥ 2, with a CAPS-IV B2 (frequency and intensity for the last week) score ≥ 5
- Men and women between 18 and 65 years of age
- Written informed consent
- The patient has the capacity to give consent (He/she is able to understand the nature and anticipated effects/side effects of the proposed medical intervention)
- The patient is not breastfeeding
- Women of child-bearing potential must have a negative urine or serum pregnancy test
- All participants must use highly effective contraception
- The patient received stable pharmacological medication for at least 4 weeks prior to study entry (any changes in medication dose or frequency of therapy must be answered with no)

2.2.5 Exclusion criteria

- Lifetime cannabis use disorder
- Current substance/alcohol use disorder (≤ 3 months);
- Acute suicidality;
- Psychotic disorder;
- Bipolar disorder;
- Current anorexia nervosa;
- Current major depressive episodes and a MADRS score > 29;
- Dementia;
- Trauma-focused psychotherapy four weeks before the trial
- Initiation of sleep medication 4 weeks prior screening or initiation of alpha adrenergic agents 4 weeks prior to screening
- Acute or unstable medical illness
- Epilepsy
- Relevant heart diseases
- Known HIV- and/or active Hepatitis-B- or Hepatitis-C-infection
- current or past malignant illness
- The patient is unwilling to consent to saving, processing and propagation of pseudonymized medical data for study reasons
- Patients, who may be dependent on the sponsor, the investigator or the trial sites, have to be excluded from the trial
- The patient is legally detained in an official institution
- The patient does have a known allergy or contraindication against Dronabinol
- The patient does have clinically significant abnormalities in 12-lead ECG
- The patient does have clinically significant laboratory abnormalities
- The patient did participate in other interventional trials during the 3 months before and at the time of this trial

2.2.6 Randomization

Patients will be randomized at baseline in a 1:1 allocation using block randomization with fixed block lengths stratified by study center.

2.2.7 Sample size

The sample size calculation is based on the primary endpoint “Clinician-Administered PTSD Scale-IV (CAPS-IV) B2 score at 10 weeks” in the ITT population including all randomized patients who received the study medication at least once. The aim is to show a lower average score at 10 weeks in the intervention group (I) than in the control group (C). Sample size calculation will be based on a two-sided, two-sample t-test. Adjustment for baseline scores in an ANCOVA model which is applied for the primary efficacy analysis will increase the power compared with a two-sample t-test, which ignores the influence of different baseline values. Therefore, this strategy for sample size calculation is a conservative procedure. In a recent random-effects meta-analysis of four RCTs investigating the effect of prazosin on PTSD nightmares, a combined standardized mean difference (Cohen’s d) of 0.5 (95% CI = 0.03–0.96) was observed (Augedal, Hansen, Kronhaug, Harvey, & Pallesen, 2013). We conservatively assume a standardized effect which is a little smaller and given by 0.45. This assumed effect can be considered as clinically relevant because distressing nightmares are an independent risk factor for comorbidity and severity in PTSD patients. Nightmares also contribute to alcohol and substance abuse, suicidal ideation, and even completed suicide (Raskind et al., 2007). As studies assessing minimal clinically important difference (MCID) for reduction of nightmares in PTSD are currently missing, clinical significance cannot be used to calculate sample size.
The required sample size to detect this effect of 0.45 with a power of 0.8 is 158 (79 per group) calculated with nQuery Version 8.2.1.0. We conservatively assume that there will be no more than 10% of patients who are randomized but never get the study medication. Therefore the total number of patients to be recruited is 176 (158/176 = 0.9) or 88 per group.

3 Analysis sets

3.1 Definitions

The full-analysis set consists of all randomized patients, which received the study medication at least once. Patients are analysed as randomized. This is the population as close as possible to the intention-to-treat population. Exclusion of patients who never received the study medication is explicitly stated as an option in the ICH E9 Guideline.
The safety set as the basis for all safety analyses corresponds to the full analysis set.

3.2 Application

The interim analysis will be done in the full-analysis set.

4 Trial centres

The study so far was conducted in 3 centers (two in Berlin and one in Mannheim) in Germany.

5 Analysis variables for the interim analysis

5.1 Demography and baseline characteristics

- Age [years]
- Gender
- CAPS IV B2 score at screening

5.2 Primary variable

Frequency and intensity of nightmares, measured with the Clinician-Administered PTSD Scale (CAPS-IV) B2 score for the last week, range 0–8 (Blake et al., 1995) directly after last intervention (10 weeks).

Due to the very short-lived effect of the drug, we will further investigate the CAPS-IV B2 score at the last visit under medication. This is either after the last intervention (10 weeks) for study completers or throughout the trial if the patient stopped taking medication. If the stop occurs in the dosing phase, the last visit before the stop will be taken as the measure.

5.3 Secondary variables

- Change from baseline of the CAPS-5 total score (overall PTSD symptoms, last week) at Visit 6 and Visit 8

6 Handling of missing values and outliers

6.1 Missing values

Missing values for the analysis of the primary endpoint will be imputed as follows: If the follow-up assessment at 10 weeks is missing but the patient has at least one follow-up assessment after baseline, last observation carried forward will be used for missing value imputation. If no value post-baseline is available, the patient will be excluded from the analysis.

If the baseline visit is missing but follow-up assessments are available, the baseline value will be imputed using multiple imputation.

6.2 Outliers

No outlier detection will be done in the interim analysis.

7 Statistical analyses / methods

7.1 General remarks

Descriptive analyses for continuous variables includes number of available data including percentage, mean, standard deviation, median, 25th percentile, 75th percentile, minimum, and maximum. For categorical variables, it includes number of available data including percentage, absolute and relative frequency. In general, results will be shown for the whole cohort and per treatment group.

7.2 Demography and baseline characteristics

Demographic and baseline variables will be analysed descriptively.

7.3 Primary analysis

We use the conditional rejection principle for the interim analysis in order to account for the unplanned analysis.

The overall one-sided alpha will be 0.025 (which is equivalent to the initially planned two-sided alpha of 0.05 but a two sided calculation is not possible due to the non-binding futility boundary).

The IA will be done after 79/176 (44.9%) patients finished visit 8 (79 is half of the needed analyzable patients (158) since currently there are no non-starters - the initial reason for an increased recruitment number)

For the boundaries we choose alpha_1 equal to 0.0008213 which is according to an alpha spending scheme (O´Brien Fleming) with 2 looks and a non-binding futility alpha_0 of 0.5 (indicating efficacy in the wrong direction).

For the effect size, the following formula given in Nakagawa and Cuthill will be used:

$d = t^{*}\top(n_1 + n_2) / \surd(n_1{*}n_2{*}df)$,

where d is the equivalent of Cohen's d for an adjusted regression coefficient, t is the t-test statistic from the adjusted regression coefficient, n_1 and n_2 are the sample sizes for the treatment and the control group, and df is the number of degrees of freedom from the adjusted regression model. The adjusted regression model will include the CAPS IV at week 10 as dependent variable and the baseline CAPS IV, the center and the treatment group as independent variables. The t statistic from the regression coefficient for the treatment group will be used for the calculation of d.

If the interim analysis indicates continuation, a sample size recalculation will be done using the previously assumed effect size of Cohen's d = 0.45. If the recalculated sample size exceeds an additional number of 80 patients beyond the initially planned 176 patients, a conditional power calculation for a design with n = 256 (176+80) will be done.

If the conditional power is less than 50%, the trial will also be stopped due to futility.

The analogous regression model for the last CAPS IV B2 under medication as dependent variable will be calculated as part of the sensitivity analysis.

7.4 Secondary analyses

7.4.1 Secondary endpoints

The CAPS IV B2 at baseline and the differences from baseline to visits 6 and from baseline to visit 8 will be shown descriptively.

7.4.2 Adverse events

Adverse events will be listed per patient. The number of Adverse events (AE) and serious adverse events (SAE), number of AES and SAE that led to discontinuation and shown descriptively. The number of AEs and SAEs in each severity and relationship to treatment category will also be shown. The same will be done for the number of patients with AEs and SAEs.

8 Software

For the analysis the software R (version 4.1.2 or newer) will be used.

9 References

Nakagawa, S. and Cuthill, I.C. (2007), Effect size, confidence interval and statistical significance: a practical guide for biologists. Biological Reviews, 82: 591-605. https://doi.org/10.1111/j.1469-185X.2007.00027.x
